# Need for cognitive closure predicts preference for similar others and reduced diversity in social networks

**DOI:** 10.1038/s41598-026-36288-6

**Published:** 2026-01-16

**Authors:** Katarzyna Growiec, Ewa Szumowska

**Affiliations:** 1 Institute of Psychology, SWPS University, ul. Chodakowska 19/31, Warsaw, 03-815 Poland; 2 Institute of Psychology, Jagiellonian University in Kraków, ul. Ingardena 6, Kraków, 30-060 Poland

**Keywords:** Heterophilous interactions, Homophilous interactions, Social networks, Need for cognitive closure, Uncertainty reduction, Psychology, Human behaviour

## Abstract

People vary in their tolerance for uncertainty, and this motivation—known as Need for Cognitive Closure (NFC)—can shape social behavior. Individuals high in NFC are driven to avoid ambiguity and quickly resolve uncertainty. Because interacting with similar others (homophily) tends to reduce uncertainty, while engaging with dissimilar others (heterophily) can increase it, we examined whether NFC predicts preferences for homophilous over heterophilous interactions. Across four correlational studies and a preregistered experiment, we found that higher NFC was consistently associated with lower heterophily. Meta-analytic results confirmed the robustness of this association across samples. High-NFC individuals also reported fewer acquaintances and fewer people to discuss important matters with. To investigate whether uncertainty reduction motivates these patterns, we experimentally induced uncertainty and assessed participants’ social preferences. As predicted, uncertainty reduced interest in interacting with dissimilar others, especially among individuals high in NFC. These findings suggest that a fundamental motivation to reduce uncertainty influences the diversity and composition of people’s social networks. This has broader implications for social support, the construction of shared realities, and the persistence of group-based stereotypes.

**Supplementary Information:**

The online version contains supplementary material available at 10.1038/s41598-026-36288-6.

## Introduction

People are embedded in intricate social networks that unfold in contexts often marked by ambiguity, complexity, and unpredictability. To manage this uncertainty, individuals tend to simplify their social worlds by forming ties with a limited number of others or only with those who are similar, rather than dissimilar to them. However, in so doing they may sacrifice some of the benefits of a richer and more diverse social network.

This tension has led scholars to pay greater attention to homophilous and heterophilous interactions. Heterophilous interactions describe relations between two actors who differ in key attributes—such as social background, beliefs, or lifestyles—whereas homophilous interactions occur between actors who are similar along these dimensions.

Although engagement in homophilous or heterophilous interactions is often structured by social opportunities^1^, it may also depend on individuals’ motivational preferences and dispositions. People differ in their tolerance for ambiguity, uncertainty, and unpredictability. While some welcome complexity and novelty in their social lives, others prefer greater structure, predictability, and simplified, black-and-white representations of the world. This differentiation is well captured by individual differences in the need for cognitive closure (NFC)^2^—a basic motivational tendency to avoid and quickly reduce uncertainty and resolve ambiguity, reflecting the desire for “an answer on a given topic, any answer. compared to confusion and ambiguity” [2 p. 337].

We therefore hypothesize that NFC is associated with the social interactions people form. Specifically, we expect high NFC to be negatively related to heterophilous interactions.

Our paper thus contributes to understanding the psychological factors—beyond personality traits—that shape the emergence and maintenance of one’s social ties. This, in turn, has implications for subjective well-being^3,4^, career and mobility outcomes^5–7^, economic performance^8,9^, mental and physical health^10,11^ and social and political beliefs^12^.

### Heterophilous and homophilous interactions

Social networks create opportunity structures and provide people with various resources^9,13,14^. These resources, however, depend on the types of interactions, mainly heterophilous and homophilous ones. Homophilous and heterophilous interactions are defined in terms of attributes of individuals involved in an interaction. Rogers and Bhowmik^15^ state that homophilous interactions refer to similarity with respect to certain attributes––such as beliefs, values, education, or social status––between pairs of individuals who interact. Heterophilous interactions are the converse, involving individuals who differ in such attributes. “More specifically, you count all the ties where ego and alters all share the same attribute, and divide this number by all the ties found in the ego network. If the result is 1, then all ties are homophilous ties. Any number below 1 can be seen as the portion of ties that are homophilous” [16 p.130]. The remaining ties are heterophilous ones.

According to Lin^17^, homophilous interactions are normative and ordinary, whereas heterophilous interactions are nonnormative and often provide access to novel information and opportunities. Numerous studies show that individuals indeed prefer to form social ties with similar others—a phenomenon known as the *like-me hypothesis* or *homophily principle*^18–20^. This tendency aligns with Donn Byrne’s reinforcement model of attraction^21–23^, which posits that people are motivated to maintain a coherent and consistent view of the world—an effectance motive. Agreement from similar others validates one’s attitudes and beliefs, reinforcing a sense of cognitive consistency and producing positive affect, whereas disagreement introduces inconsistency and anxiety, reducing attraction^24^.

Heterophilous interactions are associated with this discomfort and demand more effort, as interacting partners must navigate differences in social position, cultural background or norms–and, on top of that, assess each other’s willingness to engage in social exchange^25,26^. Such ties also tend to weaken or dissolve more quickly than homophilous ones because they are less embedded within social structures^27^. However, homophilous interactions alone are “insufficient to explain the variety of social relationships that exist”^28^, p. 58]. Some individuals deliberately seek ties with dissimilar others, gaining access to nonredundant information and novel opportunities^9,17,29^. These individuals benefit from greater exposure to diverse perspectives and resources, which can promote learning, adaptability, and innovation^17,29^.

Homophilous interactions primarily satisfy the safety drive, offering predictability and emotional support that helps maintain the status quo^13,24^. In contrast, ties with dissimilar others serve the effectiveness drive, broadening access to information and promoting innovation^13,30,31^. Rather than representing two distinct forms of relationships, these tendencies reflect opposite poles on a single continuum of social tie composition—from homogeneous and predictable to diverse and exploratory. The following section examines how individual differences in need for cognitive closure may influence where people position themselves along this continuum of social interaction.

### Need for cognitive closure vs. heterophilous and homophilous interactions

The need for cognitive closure (NFC) reflects stable individual differences in the preference for order, predictability, and structure, as well as in tolerance for ambiguity, closed-mindedness, and decisiveness^32^. High NFC levels have been associated with pressures toward opinion uniformity, encouragement of autocratic leadership, in-group favoritism, rejection of deviants, resistance to change, conservatism, and the perpetuation of group norms – a pattern referred to as *group-centrism*^33^. Individuals high in NFC tend to focus on their in-group as the primary source of one’s social reality^34^ and prefer homogeneous over heterogeneous groups^35^. They also prefer self-similar groups that validate their own worldview^35^, striving for a “shared reality” grounded in group consensus and uniformity^33^.

This motivational pattern parallels propositions of Socioemotional Selectivity Theory^36^, which suggests that when individuals perceive their time or situational horizons as limited, they prioritize emotionally meaningful, familiar, and predictable social interactions over novelty or information seeking. Both perspectives emphasize that reduced tolerance for uncertainty—whether epistemic or temporal—drives a preference for stable, homophilous social environments.

Accordingly, high NFC individuals should be particularly motivated to maintain homophilous interactions, as social ties with close and similar others provide a uniform environment and stable worldview that gratify the epistemic need for certainty—serving as “closure providers.” Conversely, high-NFC individuals should be less willing to invest in maintaining heterophilous interactions. In fact, they might feel compelled to reduce the level of unpredictability and uncertainty brought by ties with dissimilar others.

### Hypotheses

We thus hypothesize that:

(Hypothesis 1) Need for cognitive closure (NFC) is associated with lower heterophily within social networks.

In correlational Studies 1–4, we examine heterophilous interactions by using a questionnaire-based measure. In Study 2, we additionally test whether NFC is related to individuals’ network size (degree, i.e., number of social ties). Studies 3–4 extend this approach using a scenario-based measure of social interactions, and Study 3 further examines whether these associations remain when controlling for anxiety, self-esteem, and gender—factors known to influence social tie formation^37–39^.

These four studies draw on independent samples: Study 1 includes Polish economics and business university students; Study 2 includes Polish psychology students; Study 3 includes Polish students from various majors; and Study 4 includes U.S. students. To maximize statistical power and clarity of presentation, we meta-analytically summarize the relationships between NFC and heterophilous interactions across all four studies. Supplementary variables included in individual studies are presented as additional analyses.

We complement these correlational findings with an experimental test (Study 5), which allowed us to draw causal inferences. In this study, we tested whether motivation to reduce uncertainty underlies the relationship between the need for cognitive closure and the type of social interactions people form. We thus experimentally manipulated uncertainty and predicted that:

(Hypothesis 2) Participants in the uncertainty (vs. control) condition will show a weaker preference for heterophilous interactions.

(Hypothesis 3) These effects will be moderated by dispositional NFC, such that the difference between conditions is stronger among high-NFC than low-NFC participants.

Hypotheses 2–3 were pre-registered at https://aspredicted.org/8VS_FLN.

## Results of correlational studies 1–4

### Hypotheses testing

To test our hypotheses, we conducted a series of linear regression analyses predicting the extent of heterophilous interactions from need for cognitive closure (NFC) across four independent studies. The results of these analyses are summarized in Table 1.

**Table 1:** Results of linear regression analyses predicting heterophilous interactions from Need for Cognitive Closure (NFC) across Studies 1–4.

Study no. (*N*)	**Regression results**			
	**β**	**SE**	*t*	*p*
Study 1 (*N* = 101)				
Intercept	0.03	0.09	0.32	.749
NFC	-0.13	0.10	-1.41	.161
Study 2 (*N* = 131)				
Intercept	0.00	0.08	0.03	.974
NFC	-0.36	0.08	-4.36	<.001***
Study 3 (*N* = 301)				
Intercept	0.00	0.06	0.06	1.00
NFC	-0.18	0.06	-3.17	.002**
Study 4 (*N* = 296)				
Intercept	0.12	0.04	2.63	.009**
NFC	-0.15	0.04	-3.30	.001**

*Notes:* NFC = Need for Closure.

*p < .05. ** *p* < .01. *** *p* < .001.

Across all studies, the coefficients were negative, indicating that individuals higher in NFC reported less heterophilous interactions. The association reached statistical significance in Studies 2–4, while in Study 1 the effect was in the predicted direction but not significant, possibly due to the smaller sample size.

To evaluate the robustness of this effect across studies, we computed a summary estimate using a random-effects meta-analysis implemented in the metafor R package^40^. Standardized regression coefficients (β) from the individual studies were used as effect sizes, and sampling variances were based on their reported standard errors. The random-effects model (REML estimation) yielded a significant negative association between the need for cognitive closure (NFC) and heterophilous interactions, β = − 0.20, SE = 0.05, Z = − 4.32, *p* <.001, 95% CI [–0.29, − 0.11]. The test for heterogeneity was not significant, Q(3) = 5.86, *p* =.119, and the estimated between-study variance was small (*I*² = 49.2%), indicating moderate but acceptable variability across samples.

**Fig. 1:**
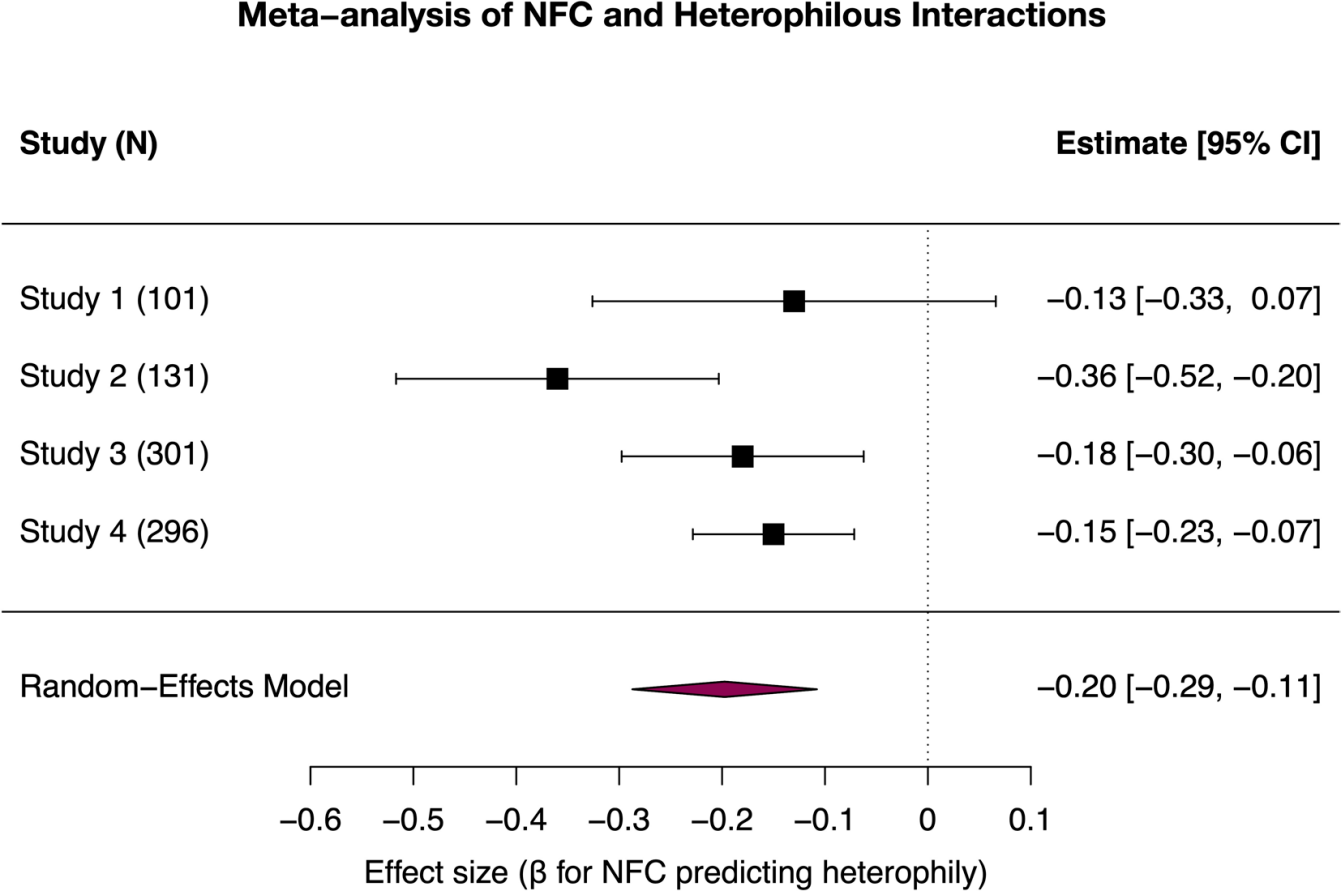
Relationship between NFC and heterophilous interactions across four studies.

These results provide convergent evidence across four independent studies that individuals higher in NFC tend to report less diverse (i.e., lower-heterophily) social interactions (see Fig. 1).

In other words, people with high levels of NFC are more likely to form ties with similar others and less likely to form ties with dissimilar others. This tendency may have important implications for creating and maintaining “shared realities”^33^, sustaining stereotypes and prejudice, and resisting belief change—due to the reinforcing influence of similar others and the lack of exposure to dissimilar perspectives.

### Additional analyses

We conducted several supplementary analyses to further assess the robustness and scope of our findings. First, we examined whether the relationship between NFC and heterophilous interactions remained significant when controlling for anxiety, self-esteem and gender—key factors previously linked to social network maintenance^39,41^. Next, we investigated whether NFC was related to social network size (i.e., the number of social ties) and social support. Finally, we explored the relationships between NFC and a scenario-based measure of social interactions, designed to capture behavioral tendencies in hypothetical social situations.

### Including statistical controls

To assess whether the association between NFC and heterophilous interactions could be explained by other individual differences, we conducted a hierarchical regression analysis including state and trait anxiety, self-esteem, and gender as covariates. In Step 1, NFC was entered as the sole predictor. In Step 2, we added state and trait anxiety; in Step 3, self-esteem; and in Step 4, gender.

Across all steps, NFC remained a significant negative predictor of heterophilous interactions, indicating that individuals with higher NFC consistently reported less diverse (lower-heterophily) social networks even after accounting for these control variables (see Supplementary Material 4 for detailed results). Thus, the relationship between NFC and social interaction diversity appears robust and independent of anxiety, self-esteem, and gender.

### Degree and social support

In Study 2, participants additionally reported the number of people they regularly socialized with during the previous month, categorized as family members, friends, and acquaintances from their workplace, school, or neighborhood. Participants also indicated how many people they had discussed important matters with during the past six months (see Table 4 for descriptive statistics and correlations with heterophilous interactions).

To examine whether network size (degree) was related to NFC, we computed Spearman’s rank correlations between NFC and the number of contacts in each category. The results showed no significant association between NFC and the number of family members (ρ = 0.07, *p* =.403) or friends (ρ = –0.14, *p* =.119) with whom participants regularly socialized. However, NFC was significantly and negatively associated with the number of acquaintances (ρ = –0.22, *p* =.013) and the number of people participants discussed important matters with (ρ = –0.22, *p* =.010).

These findings indicate that individuals high in NFC tend to have fewer peripheral social ties, particularly with acquaintances——consistent with the observed negative association between NFC and heterophilous interactions. They also reported fewer discussion partners, suggesting reduced access to social support. However, this measure reflects only the quantity of support, not its quality (individuals may maintain fewer but closer or more meaningful ties).

In sum, while NFC was unrelated to the number of close ties (family and friends), it was linked to a smaller and potentially less diverse broader network. These exploratory findings further support the notion that individuals with higher NFC engage in more selective social interactions.

### Scenario-based measure of social interactions

In Studies 3 and 4, we included an additional scenario-based measure of social interactions to assess the robustness of our findings. In this task, participants were presented with three brief scenarios describing everyday social situations—attending an annual company banquet, moving to a new neighborhood, or relocating to a new country (see Supplementary Material 3). In each scenario, they rated how likely they would be to focus on familiar social ties (homophilous interactions) or engage with new and unfamiliar people (heterophilous interactions).

We then examined the relationship between these measures and NFC. In Study 3, NFC was positively correlated with homophilous interactions (*r* =.35, *p* <.001) and negatively correlated with heterophilous interactions (*r* = –.15, *p* =.009), replicating the pattern observed for the trait-based heterophily measure. In Study 4, the corresponding correlations were *r* =.22, *p* <.001 for homophilous interactions and *r* =.05, *p* =.434 for heterophilous interactions. The latter nonsignificant correlation may reflect cross-cultural differences between the Polish (Study 3) and American (Study 4) samples, pointing to the influence of contextual and normative factors—such as greater social openness and norms encouraging engagement with diverse others in the U.S. context^42–44^. Consequently, scenario-based responses may be more sensitive to social desirability and cultural context than trait-based measures.

### Experimental study results

To test Hypotheses 2–3, we conducted multilevel analyses of the experimental data, nesting rated network members (friends and acquaintances) within participants. The dependent variable (DV) was participants’ willingness to interact with each nominated person. The independent variable (IV) was experimental condition (uncertainty vs. control), and the moderator was the similarity between the participant and each network member (higher values indicate greater similarity and thus lower heterophily). We ran separate models for demographic similarity and psychological similarity.

For all analyses, we used the *lme4* package in R^45^. To examine simple slopes and visualize the interactions, we utilized the *interactions*^46^, *sjplot*^47^ and *effects*^48^ R packages. Prior to analysis, all continuous variables were grand-mean centered. Full results for both similarity indices are detailed in Table 2.

**Table 2:** Results of multilevel analysis for demographic and psychological similarity indices.

	Demographic similarity				Psychological similarity			
Parameter	Est.	(SE)	t	*p*	Est.	(SE)	t	*p*
*β*0	−0.07	(0.06)	−1.24	0.216	−0.03	(0.05)	−0.66	0.511
*β*1 (condition)	0.17	(0.08)	1.89	0.060	0.18	(0.08)	2.27	0.024*
*β*2 (similarity)	0.13	(0.03)	5.05	< 0.001***	0.25	(0.03)	9.93	< 0.001***
*β*2 (condition × similarity)	0.10	(0.04)	2.39	0.017*	0.03	(0.04)	0.86	0.392
Individual-level random part								
$\:{{\upsigma\:}}_{\mathrm{u}0}^{2}$ (intercept variance)	0.40	(0.63)			0.32	(0.57)		
Target-level random part								
${{\sigma }}_e^2$	0.56	(0.75)			0.50	(0.71)		

Note. *p <.05. ** *p* <.01. *** *p* <.001.

Similarity emerged as a significant positive predictor of willingness to interact in both models. That is, participants were more inclined to interact with others perceived as similar to themselves on both demographic and psychological dimensions.

**Fig. 2:**
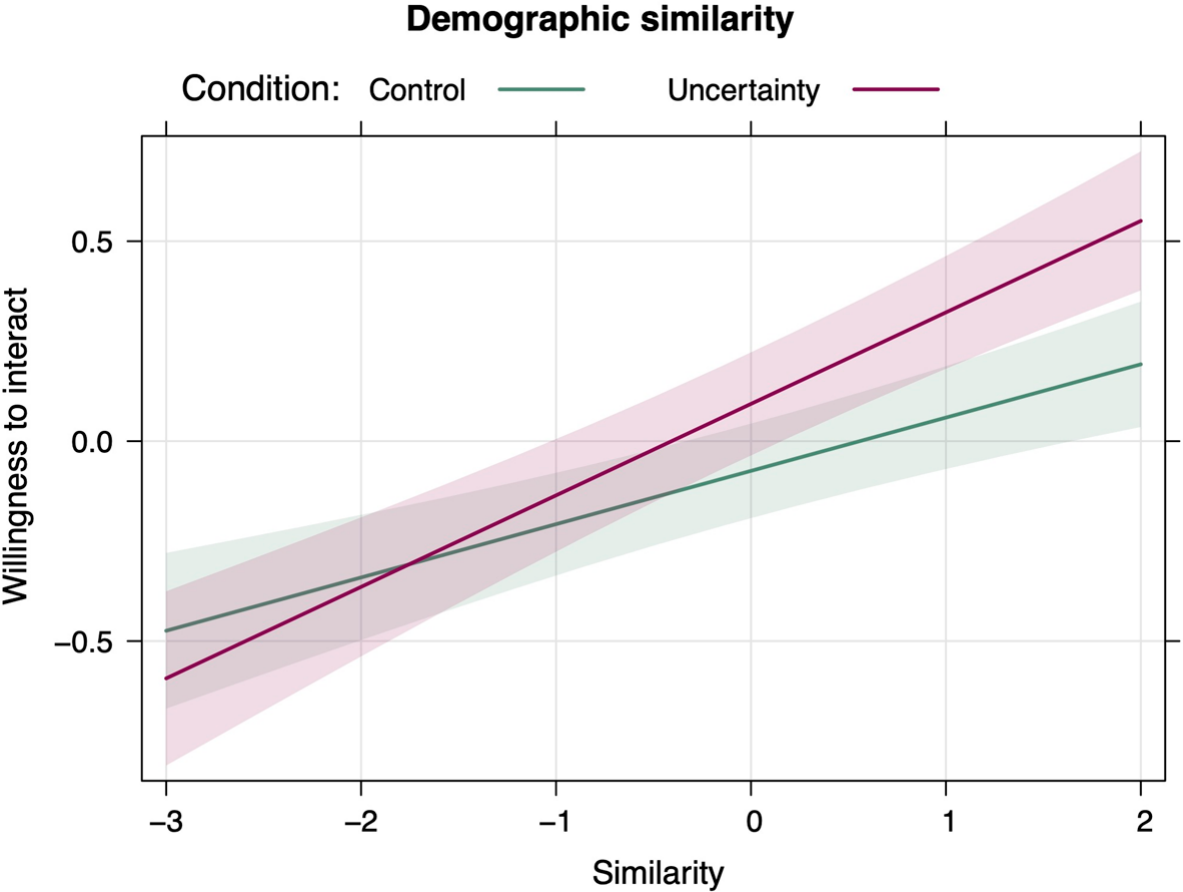
Interactive effect of condition and demographic similarity on willingness to interact.

For demographic similarity, we also observed a significant interaction with condition, indicating that although the effect of similarity was significant in both conditions, it was significantly stronger under uncertainty. Specifically, the effect of similarity on willingness to interact was equal to Est. = 0.13, SE = 0.03, *t* = 5.05, *p* <.001 in the control condition and Est. = 0.23, SE = 0.03, *t* = 7.64, *p* <.001in the uncertainty condition (see Fig. 2).

**Fig. 3:**
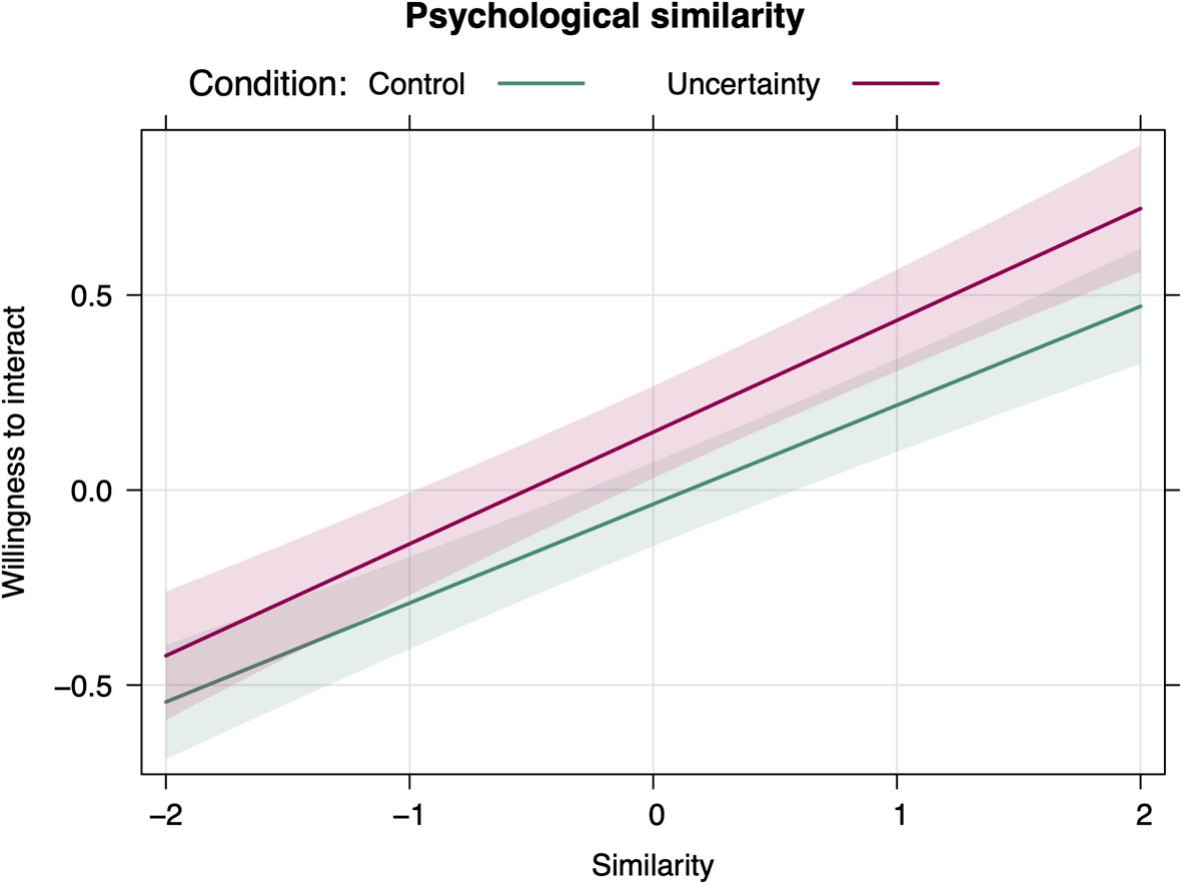
Effect of condition and psychological similarity on willingness to interact.

For psychological similarity, the interaction with condition was not significant (see Table 2). However, there was a main effect of condition, such that participants reported higher willingness to interact with others in the uncertainty compared to the control condition. The results of this analysis are presented in Fig. 3.

### Interaction with need for cognitive closure (NFC)

**Fig. 4:**
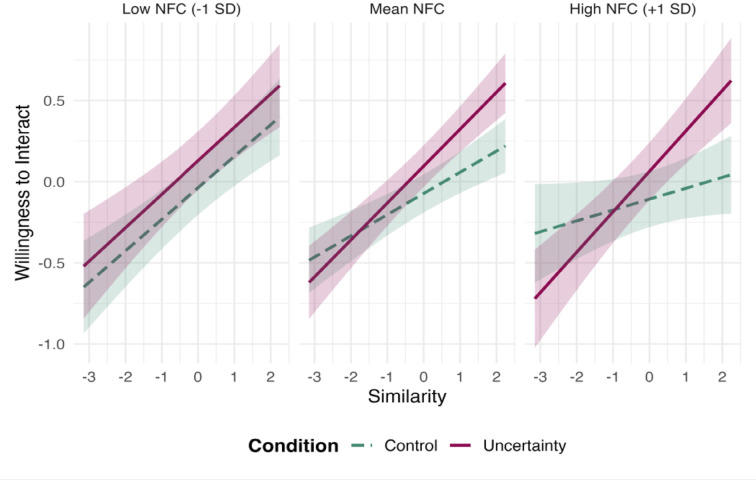
Interactive effect of condition, similarity, and need for closure on willingness to interact.

We next examined whether these effects were moderated by dispositional need for cognitive closure (NFC). To this end, we reran the models including NFC as an additional moderator. The results, presented in Table 3; Fig. 4, indicated a significant three-way interaction among condition, NFC, and demographic similarity.

Specifically, while no interaction between similarity and condition emerged among low-NFC participants, a significant effect appeared at mean and high levels of NFC. For individuals high in NFC, similarity did not influence interaction preference in the control condition, but it did under uncertainty—participants became more willing to interact with similar than dissimilar others in this condition. Simple slopes analyses (see Supplementary Material 8) confirmed that this effect of uncertainty was significant only at high levels of similarity and among participants with average or high NFC, but not at low NFC or low similarity. This pattern supports our prediction that uncertainty amplifies similarity-based preferences primarily in individuals high in need for closure.

**Table 3:** The results of multi-level analysis for models with demographic and psychological similarity indices with NFC as additional moderator.

	Demographic similarity				Psychological similarity			
Parameter	Est.	(SE)	t	*p*	Est.	(SE)	t	*p*
*β*0	−0.07	(0.06)	−1.21	0.227	−0.03	(0.05)	−0.63	0.531
*β*1 (condition)	0.17	(0.09)	1.90	0.059	0.18	(0.08)	2.26	0.025*
*β*2 (similarity)	0.13	(0.03)	4.95	< 0.001***	0.25	(0.03)	9.88	< 0.001***
*β*3 (NFC)	−0.04	(0.06)	−0.56	0.576	−0.02	(0.06)	−0.47	0.637
*β*4 (condition × similarity)	0.10	(0.04)	2.43	0.015*	0.03	(0.04)	0.84	0.402
5 (condition × NFC)	0.003	0.09	0.03	0.977	−0.04	(0.08)	−0.59	0.553
*β*6 (similarity × NFC)	−0.06	0.03	−2.26	0.024*	−0.02	(0.02)	−0.94	0.349
*β*7 (condition × similarity × NFC)	0.09	0.04	2.10	0.036*	0.04	0.03	1.12	0.262
*Individual-level random part*								
$\:{{\upsigma\:}}_{\mathrm{u}0}^{2}$ (intercept variance)	0.39	(0.63)			0.32	(0.57)		
*Target-level random part*								
${{\sigma }}_e^2$	0.55	(0.74)			0.50	(0.71)		

Note: NFC = Need for closure.

These results indicate that both situational uncertainty and dispositional need for closure jointly shape individuals’ willingness to interact across social boundaries. Under uncertainty, high-NFC individuals increasingly favor interactions with demographically similar others, reflecting an uncertainty-reduction motive.

To ensure the effects were not driven by participants’ political ideology, we conducted sensitivity analyses controlling for political orientation (categorized as right, center, or left, based on reported vote in the 2019 Polish parliamentary elections). The main effects and interactions remained significant or marginally significant across models, indicating that the observed patterns were robust beyond ideological alignment. Notably, an additional three-way interaction emerged for psychological similarity, uncertainty, and NFC, suggesting a possible suppression effect. Full model results and interpretations are presented in Supplementary Material 9.

## Discussion

This research demonstrated that the motivation to reduce uncertainty—operationalized as the need for cognitive closure (NFC)—systematically shapes people’s willingness to interact with dissimilar others. We have confirmed empirically that because high NFC individuals strive for a known, predictable, and stable world, they prefer to interact with those who are similar to themselves. Moreover, since they tend to avoid uncertainty and ambiguity, they are less inclined to interact with dissimilar others, as each new person brings some additional degree of complexity and, when not yet known well, uncertainty into the relationship—something that high-NFC individuals tend to avoid. Low-NFC individuals, on the other hand, tolerate or even seek novelty and diversity in their social ties.

Specifically, our experimental study has provided causal evidence that the motivation to reduce uncertainty is a key mechanism linking NFC to social interaction patterns. We found that under increased uncertainty, people were more willing to interact with similar others. Moreover, dispositional NFC moderated this relationship: the manipulation did not affect individuals low in NFC, but it did for those high in NFC. In this group, there was no relationship between similarity and willingness to interact in the control condition; however, under uncertainty, the preference for similar others became significant and pronounced. This indicates that similarity becomes especially salient for high-NFC individuals when they face uncertain or unpredictable contexts.

These findings align with Socioemotional Selectivity Theory^36^, which posits that when individuals perceive time or situational horizons as limited, they shift their motivational priorities from novelty and information seeking toward emotionally meaningful and predictable social bonds. Both frameworks emphasize the adaptive function of seeking stability under uncertainty—epistemic in the case of NFC, temporal in the case of SST. Thus, our results suggest that intolerance of uncertainty may produce social selectivity patterns similar to those observed across the lifespan, where individuals increasingly prefer familiar, emotionally secure relationships.

Our results also relate to the literature on NFC and group-centrism. Kruglanski et al.^33^ demonstrated that high NFC induces pressures to opinion uniformity, in-group favoritism, rejection of deviates, and the perpetuation of group norms [see also 35, 40]. They argue that this occurs because of a striving for a “shared reality.” One way to attain shared reality is through intolerance of diversity in group composition. Our studies lend support to this notion and show that greater preference for similar—and avoidance of dissimilar—others extends to everyday social interactions as well.

The regularity that high-NFC individuals have fewer ties with dissimilar others likely also underlies mechanisms by which stereotypes and prejudice are reinforced and sustained (see 35, 50]. High-NFC individuals may be motivated to avoid socializing with dissimilar others, but interacting primarily with similar others may, in turn, further narrow their perspectives and increase resistance to change.

Further research should explore the potential bidirectional relationship between NFC and social interactions. Since NFC reflects a basic motivational tendency with biological underpinnings e.g^51–53^., it may shape the social networks individuals form. Indeed, our experimental results indicate that under uncertainty, the motivation to reduce uncertainty increases preference for homophilous ties, suggesting a causal role of epistemic motivation in shaping social composition. However, social experience may also reciprocally influence NFC: individuals embedded in more restricted and homogeneous networks may develop a stronger preference for closure over time. This interpretation aligns with recent findings suggesting that epistemic motivations are not fixed dispositions but may adaptively emerge from cumulative experiences with predictable or unpredictable social environments^54^. Moreover, because high NFC is related to fewer overall interactions and narrower discussion networks, these individuals may experience lower social support, which could have implications for subjective well-being, loneliness, and resilience.

## Methods

### Participants and procedure

#### Study 1

Data were collected by the authors in paper-and-pencil form between September and October 2015 during in-class sessions. The study was approved by the SWPS University Ethical Review Board (approval no. 21/2021/2) and complied with the Declaration of Helsinki. All participants provided written informed consent prior to participation.

Participants were 102 Polish students from an economics and business major. They were given a questionnaire on paper which included Heterophilous Interaction Questionnaire (see Measures; see Supplementary Material 1^55^;, and NFC Scale^56^ see Measures; Supplementary Material 5). The final sample, due to missing data, comprised *N* = 101 subjects (54 women; *M*_age_ *=* 20.47, *SD* = 2.7).

#### Study 2

Study 2 was conducted by the authors at SWPS University (Warsaw, Poland) and Jagiellonian University in Kraków (Cracow, Poland). Data were collected online between October and December 2016 using a university-hosted survey platform. The study protocol received approval from the SWPS University Ethical Review Board (approval no. 21/2021/2). The study was conducted in accordance with the Declaration of Helsinki. Participation was voluntary and anonymous, and electronic informed consent was obtained from all participants before the study began.

Participants were 136 Polish psychology students who took part in an online study in exchange for course credits. The final sample, due to missing data, comprised *N* = 131 subjects (104 women; *M*_age_ *=* 23.73, *SD* = 4.47). Participants were first presented with the NFC scale, then the Heterophilous Interaction Questionnaire, and several questions about their social network (e.g., degree and social support).

#### Study 3

Study 3 was carried out by the authors at SWPS University (Warsaw, Poland) and Jagiellonian University in Kraków (Cracow, Poland) between December 2016 and January 2017 using an online questionnaire distributed through university-hosted platform, university mailing lists and social media. The study was reviewed and approved by the SWPS University Ethical Review Board (approval no. 21/2021/2) and conducted in accordance with the Declaration of Helsinki. Participants gave electronic informed consent prior to data collection.

Participants were 305 students who took part in an online study in exchange for credits. There were five cases with missing data. Therefore, the final sample comprised *N* = 300 subjects (262 women; *M*_age_ *=* 27.90, *SD* = 9.08). Participants were first presented with the NFC scale, then the Heterophilous Interaction Questionnaire, three scenarios to provide the behavioral tendency measure of social interaction, self-esteem measure, and the state and trait anxiety questionnaires.

#### Study 4

Study 4 was designed and implemented by the authors between November 2018 and May 2019 through the University of Maryland’s SONA participant recruitment system. Undergraduate students participated in exchange for partial course credit. The study was implemented entirely online. All procedures were approved by the University of Maryland Institutional Review Board (IRB approval no. 1345713 “Personality and Social Networks”) and were conducted in accordance with the Declaration of Helsinki. Participants provided written informed consent electronically before beginning the study. No additional parental consent was required for participants aged 17–18, as this requirement was waived by the University of Maryland IRB. All participants themselves provided informed consent.

The sample comprised *N* = 306 UMD students. There were nine cases with missing data, therefore, the final sample comprised *N* = 295 subjects (201 women; aged between 17 and 44, *M*_age_ *=* 19.87, *SD* = 2.11). Participants were first presented the NFC scale, then the Heterophilous Interaction Quesionnaire, and scenarios to provide the behavioral tendency measure of social interaction.

The data are shared in a public repository (OSF): https://osf.io/m5evt/.

### Measures

#### Heterophilous interaction questionnaire

We used a measure of trait heterogeneity within one’s network of acquaintances, the Heterophilous Interaction Questionnaire (see Supplementary Material 1^55^;. This questionnaire encompasses 13 items related to maintaining social ties with dissimilar others on important characteristics – e.g. different ethnic background, different level of education, different income, other interests, different worldview. The characteristics chosen for the questionnaire widely overlap with the list of possible similarities between interaction partners used in^57^. Each our item began with the stem “In the circle of my close acquaintances there are people who …” and ended with a different sentence completion e.g. “are much older than me” or “have different life style than me”. Participants respond on a five-point scale ranging from 1 to 5 (from “*never or almost never”* to “*very often”*). In Studies 2–4 one item was added (“*persons who have different religious views than me”*). The reliability of the scale was equal to Cronbach’s α = 0.67 in Study 1, α = 0.81 in Study 2, α = 0.82 in Study 3 and α = 0.84 in Study 4.

### Need for cognitive closure scale

In Studies 1–3, the Need for Cognitive Closure (NFC) was measured using the 32-item Need for Cognitive Closure Scale^56^; Polish adaptation^58^:. The scale includes items such as “I think that having clear rules and order at work is essential for success” and “I don’t like to go into a situation without knowing what I can expect from it”. Participants responded on a 6-point Likert scale ranging from 1 to 6 (anchored *“I definitely disagree”* and “*I definitely agree”*). A total NFC score was computed by averaging all items, with higher scores reflecting a greater need for closure. The internal consistency of the scale was satisfactory, with Cronbach’s α = 0.79 in Study 1, α = 0.85 in Study 2, and α = 0.83 in Study 3.

In Study 4, we used the 41-item revised English version of the Need for Cognitive Closure Scale^59^, which refines the Decisiveness subscale to better represent the motivational rather than ability component of closure. Responses were given on the same 6-point scale, and Cronbach’s α = 0.86.

The complete list of items used in each study can be found in Supplementary Material 5.

#### Additional measures

Because social interactions are related to anxiety, self-esteem and gender, in Study 3 we included these variables to check whether the relationship between NFC and social interactions remains significant when they are controlled for. Also, in Study 2, we included additional questions about degree (the number of ties participants have) and in Studies 3 and 4, we used an additional, scenario-based measure of social interactions patterns (separately heterophilous and homophilous).

 State-trait anxiety inventory. anxiety was measured with the State-Trait Anxiety Inventory, STAI^60^. STAI consists of 40 questions. First 20 relate to the state of anxiety experienced by an individual, the next 20 relate to the anxiety as a trait. The reliability of the scale was equal to Cronbach’s α = 0.95 for anxiety as a state α = 0.91 for anxiety as a trait.

 Self-Esteem Scale. Self-esteem was measured with Rosenberg’s Self-Esteem Scale^61^. The scale consists of 10 items with a 4-point answer scale from *“strongly agree*” to “*strongly disagree*”. Reliability of the scale was equal to Cronbach’s α = 0.89.

Questions about degree and social support. In Study 2, we asked participants four questions related to their social networks: “*Please tell us* 1) *with how many persons like acquaintances from the workplace*,* school or neighborhood you socialized regularly during the last month;* 2) *With how many persons among your friends you socialized regularly during the last month;* 3) *With how many persons among your close family members you socialized regularly during the last month;* and 4) *Looking back at last 6 months*,* please tell us*,* with how many persons you discussed matters that are important to you.”* In all cases, the answer was the raw number of persons an individual indicated.

Scenario-based measure of social interactions. To obtain a behavioral tendency measure of social interactions, we created three scenarios in which a person is asked to imagine that s/he is going to an annual company banquet (scenario 1), has moved to a new neighborhood (scenario 2) or a new country (scenario 3) (see Supplementary Material 3). In each scenario, a person can meet new people (reflecting heterophilies interactions) or focus on social ties with familiar persons (reflecting homophilous interactions). For each scenario, there were six Likert type questions (anchored 1 – “*not at all true in reference to me”* and 7 – “*entirely true in reference to me”*). Three questions referred to homophilous interactions and three to heterophilous interactions. We averaged responses to each type of interactions (9 items for each type) and obtained a behavioral tendency measure of heterophilous and homophilous interactions. An example scenario reads: “*Recently*,* you and your family have moved to a new place. You have never lived in this area. To what extent are the below statements true to you? 1. In the first place*,* I make sure that each member of my family feels good in the new place* (homophilous interactions’ item). 2. *I first want to meet new neighbors and people from the vicinity* (heterophilous interactions’ item).” The measure was included in Studies 3 and 4. Reliability of the heterophilous interactions scale was equal to Cronbach’s α = 0.75 in Study 3 and α = 0.80 in Study 4 and reliability of the homophilous interactions scale was equal to Cronbach’s α = 0.65 in Study 3 and α = 0.61 in Study 4.

All questionnaires were administered for non-commercial academic research under appropriate licenses or open-use conditions (see Supplementary Material 6 for details on instrument licensing).

Means and standard deviations for homophilous and heterophilous interactions and NFC are presented in Table 4.

### Participants of the experimental study 5

Study 5 was conducted by the authors in June 2023 and approved by the SWPS University Ethical Review Board (approval no. 16/2023). The study was carried out in accordance with the Declaration of Helsinki, and all participants provided informed online consent prior to participation.

**Table 4:** Descriptive statistics for variables describing regular socialization.

	M	SD	median	Correlations with heterophilous interactions [ρ]
family	7.97	5.95	6.00	0.07
friends	5.63	4.59	5.00	0.07
acquaintances	20.55	20.43	15.00	0.28***
people to discuss important matters with	6.11	4.43	5.00	0.22*

*Notes*: two-tailed. Correlations are Spearman’s rank correlations.

*p <.05. ** *p* <.01. *** *p* <.001.

A priori power analysis using G*Power 3.1^62^ indicated that a sample of at least 213 participants was required to detect a significant interaction effect of *f*^*2*^ = 0.05 in a regression model with three predictors (condition, NFC, and the product of the two) at 90% power (α = 0.05). To account for potential data exclusions and unreliable responding, we aimed to recruit approximately 300 participants.

Participants were registered users of an independent Polish online research panel. Initially, 629 participants began the survey, but only 312 completed it and provided at least one name per category as requested (see below). Following our preregistered exclusion criteria, 47 participants were excluded for not completing the manipulation task. Based on qualitative coding of responses, 9 participants were excluded for providing non-meaningful names or initials, and 18 participants were excluded for non-meaningful responses in the uncertainty condition (e.g., incoherent or irrelevant answers).

The final sample comprised 238 participants: 122 women, 114 men, 1 individual identifying as other, and 1 participant who did not specify their gender. Age distribution was as follows: 27 participants fell into the 18–24 age group, 32 were 25–32, 40 were 33–40, 41 were 41–50, 32 were 51–60, and 65 were 61 years or older; one participant did not report age. Participants were compensated according to the standard rates of the research panel.

### Materials and procedure of the experimental study

The study was conducted online using the Qualtrics platform. Participants were invited to take part in a study on the relationship between personality and social ties. After providing informed consent, participants completed the shortened 15-item version of the Need for Cognitive Closure scale [63 Polish adaptation in 64]. This version retains the original scale’s structure and demonstrates comparable psychometric properties to the full version^63^. Next, participants were asked to indicate the names (or nicknames/initials) of (a) three friends from their family, (b) four friends outside of their family, and (c) three acquaintances they had met during the past year. Participants were instructed that if two individuals shared the same name, an initial of the last name should be provided to ensure that each person could be identified uniquely.

Participants were then presented with the list of individuals they had provided. Using the Choice Heterophily Questionnaire (see Supplementary Material 3), they rated the characteristics of each person to assess perceived similarity to themselves.

Following this, participants completed what appeared to be an unrelated recall task, which in fact served as the uncertainty manipulation. They were randomly assigned to either the control or uncertainty condition. After the manipulation, participants completed the Positive and Negative Affect Schedule (PANAS)^65^.

In the final segment, participants were again shown the list of names they had provided earlier (presented in randomized order) and indicated their willingness to interact with each person. The study concluded with a debriefing and participant thank-you message.

### Uncertainty manipulation

To induce a momentary sense of uncertainty among participants, we employed a recall task with two distinct conditions. In the uncertainty condition, participants were prompted to recall a situation in which they experienced uncertainty. They were asked to provide a brief description of the situation and then elaborate on their thoughts, feelings, and bodily sensations experienced during that period. The instructions read: “Please describe a situation, positive or negative, when you felt uncertain. What situation was it?” After briefly describing the event, participants responded to three specific questions:1) “What did you think then? Please describe your thoughts,” 2) “What did you feel then? Please describe your feelings,” and 3) “What bodily sensations did you experience? Please describe your sensations.”

In the control condition, participants were asked to recall the most recent occasion when they watched TV or a video on a computer. The exact wording was: “Please describe a situation when you last watched TV or a video. What film, program, or show was it?” After describing the situation, participants answered the same three follow-up questions as in the uncertainty condition.

This manipulation was pilot-tested in a separate online study (*N* = 93) and shown to be effective. In that study, one group of participants (*n* = 44) recalled an uncertain event, while another (*n* = 49) recalled a neutral event (watching TV). After completing the recall task, participants rated their current emotions on a list of items adapted from PANAS^65^: *interested*,* excited*,* strong*,* distressed*,* guilty*,* scared*,* hostile*,* enthusiastic*,* proud*,* irritable*,* alert*,* ashamed*,* inspired*,* nervous*,* determined*,* attentive*,* upset*,* active*,* afraid*,* jittery*,* sad*,* uncertain* (the last two items were added for this study).

Items were presented in random order. Before analysis, we excluded participants who failed to respond to the recall task or described an uncertain or disturbing event in the control condition (final *N* = 87). We then conducted one-way ANOVAs with condition as a between-subject factor for each item. As expected, participants in the uncertainty condition reported significantly greater uncertainty (*M* = 2.73, *SD* = 1.37) than those in the control condition (*M* = 2.05, *SD* = 1.15), *F*(1, 85) = 6.27, *p* =.014. No other emotion differed significantly between groups, confirming that the manipulation specifically increased feelings of uncertainty without altering general affect. All pilot study data are publicly available via the Open Science Framework: https://osf.io/m5evt/.

### Choice heterophily questionnaire

In the experimental Study 5, we employed a universal measure of interaction diversity—a modified version of the Choice Heterophily Questionnaire developed by Antonoplis and John^66^. While the original measure focused primarily on race, we extended it to capture a broader range of social similarity dimensions relevant to the current study.

Specifically, we distinguished between demographic similarity and psychological similarity. Demographic similarity included attributes such as age, gender, nationality, sexual orientation, skin color, financial situation, attitude toward religion, and political preference during voting in 2019.

Psychological similarity, in turn, encompassed preferences for lifestyle, music, websites, newspapers, internet platforms, movies, and books. Each item was rated on a 4-point scale (1 = *the same as me*, 2 = *partly the same*, 3 = *only a few the same*, 4 = *completely different*). Participants could also select “I don’t know” or, when applicable, “This person does not [read/watch/etc.],” which were coded as missing values and excluded from analyses.

In the adapted Choice Heterophily Questionnaire (see Supplementary Material 2), participants were instructed to nominate 10 individuals representing their social network, following these guidelines: (a) three emotionally close family members (such as a mother figure, father figure, or sibling); (b) four close friends; and (c) three acquaintances met within the past year. To ensure each individual was uniquely identifiable, participants were instructed to provide initials or nicknames if duplicate first names occurred. After nominating these individuals, participants rated 15 characteristics (e.g., age, ethnicity, gender) for each person and subsequently provided their own demographic information (see Supplementary Material 7 for detailed sample composition).

Family members were included in the nomination task because they typically constitute part of people’s social networks. However, following Antonoplis and John^66^ and prior literature^20^, family members were excluded from the analysis, as these ties are generally not freely chosen. Consequently, analyses were based on seven network members per participant (four stable friends and three new acquaintances).

Based on participants’ responses, we computed two indices of similarity. The demographic similarity score was calculated by summing matches between participants and each nominated person across the demographic criteria (age, gender, nationality, sexual orientation, skin color, financial situation, attitude toward religion, and political preference). Each dimension was coded 0 = dissimilar, 1 = similar, and then summed to yield an overall index per network member. Higher scores indicated greater demographic similarity (i.e., lower heterophily).

The psychological similarity score was computed by averaging participants’ ratings on the attitudinal and preference dimensions (lifestyle, music taste, preference for websites, newspapers, internet platforms, movies, and books). We recoded the variables so that a higher score indicates greater similarity. The resulting mean scores reflected each network member’s perceived psychological similarity to the participant, with higher values indicating greater similarity.

In accordance with our pre-registration protocol, we analyzed the demographic and psychological similarity indices separately.

### Willingness to interact

After completing the recall task, participants answered a series of questions assessing their willingness to interact with the network members they had listed earlier in the study. Specifically, we presented them with the names they provided (individually, in a random order), and asked to rate each network member on the following dimensions: “To what extent would you like to 1) meet this person, 2) talk to this person, 3) write to this person, and 4) spend time with this person.”

Responses were provided on a 7-point Likert scale (1 = *not at all*, 7 = *very much*). The four items were averaged to create a composite index reflecting each participant’s current willingness to interact with a given network member. Separate indices were computed for each individual named by the participant.

## Supplementary Information

Below is the link to the electronic supplementary material.

Supplementary Material 1

Supplementary Material 2

Supplementary Material 3

Supplementary Material 4

Supplementary Material 5

Supplementary Material 6

Supplementary Material 7

Supplementary Material 8

Supplementary Material 9

Supplementary Material 10

## Data Availability

All data supporting the findings of these studies are available in Open Science Framework at [https://osf.io/m5evt/](https:/osf.io/m5evt) The dataset is original and has not been used or published in any previous scientific article.
