# Supplementary material for: Need for cognitive closure predicts preference for similar others and reduced diversity in social networks
Source: Sci Rep. 2026 Jan 16;16:5582. doi: 10.1038/s41598-026-36288-6 (PMC12891588; doi:10.1038/s41598-026-36288-6)
Supplement: Supplementary file 1 — Supplementary Material 1 [file 41598_2026_36288_MOESM1_ESM.docx]

# Supplementary Material 1

**Heterophilous Interaction Questionnaire**

We would like to get to know something about your close acquaintances. Below you find a list of statements beginning with: “In the circle of my close acquaintance there are persons who….” Please give your answer by each statement showing how much it is true according to you. Answers are as follows:

1 – never or almost never

2 – very rarely

3 – rarely

4 – often

5 – very often

| **In the circle of my close acquaintances they are persons who** | |  |
| --- | --- | --- |
| 1. | Are much older than me | 1 2 3 4 5 |
| 2. | Have different life style than me | 1 2 3 4 5 |
| 3. | Have different nationality than me | 1 2 3 4 5 |
| 4. | Have different sexual orientation than me | 1 2 3 4 5 |
| 5. | Watch different TV channels, platforms, programs etc. | 1 2 3 4 5 |
| 6. | Are not from my “pack” from secondary school | 1 2 3 4 5 |
| 7. | Have opposite sex than me | 1 2 3 4 5 |
| 8. | Listen to different kinds of music than me | 1 2 3 4 5 |
| 9. | Like different writers than me | 1 2 3 4 5 |
| 10. | Read different newspapers and magazines than I do | 1 2 3 4 5 |
| 11. | Have different skin colour than me | 1 2 3 4 5 |
| 12. | Are much poorer than me | 1 2 3 4 5 |
| 13. | Have different political opinions than me | 1 2 3 4 5 |
| 14. | Have different religious views than me* | 1 2 3 4 5 |

* In Studies 2-4 additionally this item was used
