# Supplementary material for: Need for cognitive closure predicts preference for similar others and reduced diversity in social networks
Source: Sci Rep. 2026 Jan 16;16:5582. doi: 10.1038/s41598-026-36288-6 (PMC12891588; doi:10.1038/s41598-026-36288-6)
Supplement: Supplementary file 2 — Supplementary Material 2 [file 41598_2026_36288_MOESM2_ESM.docx]

**SM 2. Choice Heterophily Questionnaire**

Identify three emotionally closest individuals among your family.

1. ……
2. ……
3. ……

Identify four your closest friends. Identify individuals who are not part of your family.

Friends:

1. ……
2. ……
3. ……

4. ……

Then indicate three new acquaintances you have met in the last 12 months. By 'acquaintances,' we mean individuals with whom you maintain fairly regular contact (in person, by phone, or online), and both of you consider that there is a relationship between you. Provide their names, initials, or nicknames so that it is clear to you who they are:

Acquintances:

1. …...

2. …...

3. …...

Now, for each of the indicated 7 individuals, specify the following characteristics:

- Age range: 18-24; 25-32; 33-40; 41-50; 51-60; 61 and more
- Gender: female, male, other, declined to answer
- Nationality: Polish or other – please specify……….
- Sexual orientation: heterosexual, homosexual, bisexual, other, I don't know
- Skin color: light (white, pink), medium (tan, olive), dark (light brown), very dark (dark brown, black)
- Religion: Catholic, Orthodox, Protestant, Islamic, Jewish, Buddhist, Hindu, other, atheist, I don't know
- Financial situation: poor - insufficient funds for basic needs; fairly poor - need to monitor every expense; average - no problems covering current expenses; good - able to afford many things while maintaining some savings; very good - able to live in luxurious conditions; I don't know/prefer not to answer
- Attitude towards religion: religion is very important in their life; religion is rather important in their life; religion is not very important in their life; they are atheist; they are agnostic; other; I don't know
- Political party preference in the last parliamentary elections in 2019: PSL; PiS; SLD; KWiN (Confederation Freedom and Independence); KO (Civic Coalition); I don't know; didn't vote in those elections
- - Lifestyle: very similar to mine; somewhat similar to mine; not very similar to mine; completely different
- Music taste: very similar to mine; somewhat similar to mine; not very similar to mine; completely different
- Does this person read the same websites, forums, newspapers as you? - reads the same websites, forums, and newspapers as me; reads partially the same websites, forums, and newspapers as me; reads very few of the same websites, forums, and newspapers as me; reads completely different websites, forums, and newspapers than me; I don't know; this person doesn't use websites, forums, and newspapers
- Does this person use the same social media platforms as you? - uses the same ones as me; uses partially the same ones as me; uses very few of the same ones as me; uses completely different ones than me; I don't know; this person doesn't use social media
- Does this person watch the same series, vlogs, and movies as you? - watches the same ones as me; partially watches the same ones as me; watches very few of the same ones as me; watches completely different ones than me; I don't know; this person doesn't watch series, vlogs, or movies
- Does this person read the same books as you? - reads the same ones as me; partially reads the same ones as me; reads very few of the same ones as me; reads completely different ones than me; I don't know; this person doesn't read books

**Afterwards the respondent answer all these questions about herself**
