# Supplementary material for: Need for cognitive closure predicts preference for similar others and reduced diversity in social networks
Source: Sci Rep. 2026 Jan 16;16:5582. doi: 10.1038/s41598-026-36288-6 (PMC12891588; doi:10.1038/s41598-026-36288-6)
Supplement: Supplementary file 3 — Supplementary Material 3 [file 41598_2026_36288_MOESM3_ESM.docx]

# Supplementary Material 3

# Scenario-based measure of social interactions used in Study 3 & 4

Now we will present you with several scenarios. Please try to imagine each of the situations and indicate how you would behave in each of them. You answer by selecting one of the options on a given 1-7 scale wherein 1 indicates “*not at all true in reference to me”* and 7 “*entirely true in reference to me”.*

**Scenario 1**

You are going to an annual banquet organized by your company. You have been working in this company only for three months and this is the first time you are taking part in such a banquet. (*You have been working in this company for three years and this is the third time you are taking part in such a banquet*.) Your invitation comprises two people so you are taking your partner/spouse with you. How true are the below statements in reference to you?

At the banquet:

1. First of all, I am trying to meet new people. (heterophilous interaction)
2. First of all, I focus on my partner/spouse and make sure she/he feels OK. (homophilous interaction)
3. I spend most of my time talking to my partner. (homophilous interaction)
4. Such parties are a great opportunity to make new acquaintances and get to know people whom you only fleetingly see at work. (heterophilous interaction)
5. I try to talk to colleagues with whom I usually do not have the time to talk to. (heterophilous interaction)
6. I feel relieved that I could come to this party with my partner. (homophilous interaction)

**Scenario 2**

Recently, you and your family have moved to a new place. You have never lived in this area. (*You lived in this area several years ago.*) To what extent are the below statements true in reference to you?

1. In the first place, I make sure that each member of my family feels good in the new place. (homophilous interaction)
2. I first want to meet new neighbors and people from the vicinity. (heterophilous interaction)
3. If I get to know my neighbors, it will be by chance; I would rather not impose. (homophilous interaction)
4. Several days after my arrival, I am taking a welcoming gift to my neighbors (e.g., a cake, flower, a bottle of good wine) to establish good relationships. (heterophilous interaction)
5. I am happy about having moved to a new place mainly because me and my family can calmly live away from old acquaintances. (homophilous interaction)
6. It is worth to have good relations and wide contacts with various people. (heterophilous interaction)

**Scenario 3**

Because of professional reasons you moved to another country. You do not know it very well – you have never had a chance to spend more than a few days in this country. (*You lived in this country for several months during your abroad internship you completed some time ago.*) You know that your distal family lives there. Because of the distance, you have not seen them very often. How true are the below statements in reference to you?

1. I first focus on meeting my new neighbors and work colleagues and then, if I have time, I visit my family. (heterophilous interaction)
2. If possible, I want to visit my family right after my arrival. (homophilous interaction)
3. I am very happy I have “my own” in a foreign country. (homophilous interaction)
4. I am very happy I can focus on building new relations and start “anew.” (heterophilous interaction)
5. I take care about relations with my family and am trying to make up it up for not having earlier the chance to meet so often. (homophilous interaction)
6. I really want to maximally use the experience of living abroad and meet as many different people as possible. (heterophilous interaction)
