# Supplementary material for: Need for cognitive closure predicts preference for similar others and reduced diversity in social networks
Source: Sci Rep. 2026 Jan 16;16:5582. doi: 10.1038/s41598-026-36288-6 (PMC12891588; doi:10.1038/s41598-026-36288-6)
Supplement: Supplementary file 4 — Supplementary Material 4 [file 41598_2026_36288_MOESM4_ESM.docx]

Supplementary Material 4

| Table | | | | | | | | | | |
| --- | --- | --- | --- | --- | --- | --- | --- | --- | --- | --- |
| *Hierarchical regression analyses for heterophilous interactions and homophilous interactions with statistical controls (Study 3).* | | | | | | | | | | |
|  | **Heterophilous interactions** | | | | | **Homophilous interactions** | | | | |
| Variable | B | *b(se)* | β | *p* | ΔR^2^ | B | *b(se)* | β | *p* | ΔR^2^ |
| Step 1 | | | |  | .08*** |  |  |  |  | .02* |
| NFC | -5.53 | 1.10 | -.28*** | .00 |  | 2.58 | 1.12 | 0.13* | .02 |  |
| Step 2 | | | |  | .01 |  |  |  |  | .01 |
| NFC | -5.56 | 1.17 | -.18*** | .00 |  | 2.43 | 1.18 | 0.13* | .04 |  |
| State-anxiety | -0.08 | 0.06 | -.10 | .20 |  | -0.08 | 0.06 | -0.10 | .19 |  |
| Trait-anxiety | 0.06 | 0.07 | .07 | .36 |  | 0.08 | 0.07 | 0.09 | .25 |  |
| Step 3 | | | |  | .00 |  |  |  |  | .00 |
| NFC | -5.58 | 1.17 | -.28*** | .00 |  | 2.44 | 1.19 | 0.13* | .04 |  |
| State-anxiety | -0.08 | 0.06 | -.11 | .17 |  | -0.08 | 0.06 | -0.10 | .22 |  |
| Trait-anxiety | 0.03 | 0.08 | .04 | .69 |  | 0.10 | 0.08 | 0.11 | .22 |  |
| Self-esteem | 0.97 | 1.13 | .06 | .39 |  | -0.47 | 1.15 | -0.03 | .68 |  |
| Step 4 | | | |  | .02* |  |  |  |  | .01 |
| NFC | -5.76 | 1.16 | -.29*** | .00 |  | 2.31 | 1.18 | 0.12* | .05 |  |
| State-anxiety | -0.09 | 0.06 | -.11 | .14 |  | -0.08 | 0.06 | -0.10 | .19 |  |
| Trait-anxiety | 0.04 | 0.08 | .04 | .63 |  | 0.10 | 0.08 | 0.12 | .20 |  |
| Self-esteem | 1.19 | 1.13 | .08 | .29 |  | -0.31 | 1.15 | -0.02 | .79 |  |
| Gender (ref. cat.: male) | -3.10 | 1.38 | -.13* | .04 |  | -2.33 | 1.41 | -0.10 | .10* |  |
