# Supplementary material for: Need for cognitive closure predicts preference for similar others and reduced diversity in social networks
Source: Sci Rep. 2026 Jan 16;16:5582. doi: 10.1038/s41598-026-36288-6 (PMC12891588; doi:10.1038/s41598-026-36288-6)
Supplement: Supplementary file 5 — Supplementary Material 5 [file 41598_2026_36288_MOESM5_ESM.docx]

**Supplementary Material 5**

**NEED FOR COGNITIVE CLOSURE SCALE**

(Webster & Kruglanski, 1994; Revised by Roets & Van Hiel, 2007)

*Please read the following statements carefully. For each one, select the response that best expresses your opinion. Make sure you mark your answer next to the correct statement. Please respond to all statements.*

1 – strongly disagree
2 – disagree
3 – somewhat disagree
4 – somewhat agree
5 – agree
6 – strongly agree

I think that having clear rules and order at work is essential for success (1)

Even after I've made up my mind about something, I am always eager to consider a different opinion (2)

I don't like situations that are uncertain (3)

I dislike questions which could be answered in many different ways (4)

I like to have friends who are unpredictable (5)

I find that a well-ordered life with regular hours suits my temperament (6)

When dining out, I like to go to places where I have been before so that I know what to expect (7)

I feel uncomfortable when I don't understand the reason why an event occurred in my life (8)

I feel irritated when one person disagrees with what everyone else in a group believes (9)

I hate to change my plans at the last minute (10)

I don't like to go into a situation without knowing what I can expect from it (11)

When I have made a decision, I feel relieved (12)

When I am confronted with a problem, I’m dying to reach a solution very quickly (13)

When I am confused about an important issue, I feel very upset (14)

I would quickly become impatient and irritated if I would not find a solution to a problem immediately (15)

I would rather make a decision quickly than sleep over it (16)

Even if I get a lot of time to make a decision, I still feel compelled to decide quickly (17)

I think it is fun to change my plans at the last moment (18)

I enjoy the uncertainty of going into a new situation without knowing what might happen (19)

My personal space is usually messy and disorganized (20)

In most social conflicts, I can easily see which side is right and which is wrong (21)

I almost always feel hurried to reach a decision, even when there is no reason to do so (22)

I believe that orderliness and organization are among the most important characteristics of a good student (23)

When considering most conflict situations, I can usually see how both sides could be right. (24)

I don't like to be with people who are capable of unexpected actions (25)

I prefer to socialize with familiar friends because I know what to expect from them (26)

I think that I would learn best in a class that lacks clearly stated objectives and requirements (27)

When thinking about a problem, I consider as many different opinions on the issue as possible (28)

I like to know what people are thinking all the time (29)

I dislike it when a person's statement could mean many different things (30)

It's annoying to listen to someone who cannot seem to make up his or her mind (31)

I find that establishing a consistent routine enables me to enjoy life more (32)

I enjoy having a clear and structured mode of life (33)

I prefer interacting with people whose opinions are very different from my own (34)

I like to have a place for everything and everything in its place (35)

I feel uncomfortable when someone's meaning or intention is unclear to me (36)

I always see many possible solutions to problems I face (37)

I'd rather know bad news than stay in a state of uncertainty (38)

I do not usually consult many different opinions before forming my own view (39)

I dislike unpredictable situations (40)

I dislike the routine aspects of my work (studies) (41)

**SHORT NEED FOR COGNITIVE CLOSURE SCALE**

(Roets & Van Hiel, 2011)

*Please read the following statements carefully. For each one, select the response that best expresses your opinion. Make sure you mark your answer next to the correct statement. Please respond to all statements.*

1 – strongly disagree
2 – disagree
3 – somewhat disagree
4 – somewhat agree
5 – agree
6 – strongly agree

| 1 | I find that a well-ordered life with regular hours suits my temperament. |
| --- | --- |
| 2 | I dislike unpredictable situations. |
| 3 | When I am confronted with a problem, I’m dying to reach a solution very quickly. |
| 4 | I feel uncomfortable when I don’t understand the reason why an event occurred in my life. |
| 5 | I feel irritated when one person disagrees with what everyone else in a group believes. |
| 6 | I find that establishing a consistent routine enables me to enjoy life more. |
| 7 | I don’t like to be with people who are capable of unexpected actions. |
| 8 | I would quickly become impatient and irritated if I would not find a solution to a problem immediately. |
| 9 | I dislike it when a person’s statement could mean many different things. |
| 10 | I don’t usually consult many different opinions before forming my own view. |
| 11 | I enjoy having a clear and structured mode of life. |
| 12 | I don’t like situations that are uncertain. |
| 13 | When I have made a decision, I feel relieved. |
| 14 | I feel uncomfortable when someone’s meaning or intention is unclear to me. |
| 15 | I dislike questions which could be answered in many different ways. |

**SKALA POTRZEBY POZNAWCZEGO DOMKNIĘCIA**(Webster & Kruglanski, 1994; Polish adaptation by Kossowska, 2003)

*Uważnie przeczytaj poniższe twierdzenia. Przy każdym zdaniu zaznacz odpowiedź, która najlepiej wyraża Twoją opinię. Upewnij się, czy swoją odpowiedź zaznaczasz przy odpowiednim twierdzeniu. Proszę ustosunkować się do wszystkich twierdzeń.*

1 - zdecydowanie nie zgadzam się

2 - nie zgadzam się

3 - raczej nie zgadzam się

4 - raczej się zgadzam

5 - zgadzam się

6 - zdecydowanie się zgadzam

| Myślę, że aby osiągnąć sukces w pracy zawodowej, konieczne jest posiadanie jasnych reguł i ich przestrzeganie. | 1 | 2 | 3 | 4 | 5 | 6 |
| --- | --- | --- | --- | --- | --- | --- |
| Zwykle biorę pod uwagę różne opinie na temat danego zjawiska, nawet wówczas, gdy mam już wyrobiony pogląd. | 1 | 2 | 3 | 4 | 5 | 6 |
| Unikam niejasnych sytuacji. | 1 | 2 | 3 | 4 | 5 | 6 |
| Unikam stawiania pytań, na które można odpowiedzieć na wiele różnych sposobów. | 1 | 2 | 3 | 4 | 5 | 6 |
| Cenię sobie przyjaciół, którzy są nieprzewidywalni. | 1 | 2 | 3 | 4 | 5 | 6 |
| Myślę, że dobrze uporządkowane życie jest zgodne z moim temperamentem. | 1 | 2 | 3 | 4 | 5 | 6 |
| Zawsze, kiedy idę zjeść coś na mieście, wybieram miejsce, w którym wcześniej byłem/am i wiem, czego można się tam spodziewać. | 1 | 2 | 3 | 4 | 5 | 6 |
| Czuję się źle, kiedy nie rozumiem powodów, dla których pewne sytuacje zdarzają się w moim życiu. | 1 | 2 | 3 | 4 | 5 | 6 |
| Unikam brania udziału w wydarzeniach, nie wiedząc czego mogę się po nich spodziewać. | 1 | 2 | 3 | 4 | 5 | 6 |
| Po podjęciu decyzji odczuwam ulgę. | 1 | 2 | 3 | 4 | 5 | 6 |
| Gdy staję przed jakimś problemem, za wszelką cenę chcę go bardzo szybko rozwiązać. | 1 | 2 | 3 | 4 | 5 | 6 |
| Źle się czuję, jeżeli nie mam pewności, co myśleć lub jak się zachować w jakiejś ważnej sprawie. | 1 | 2 | 3 | 4 | 5 | 6 |
| Jeśli nie mogę znaleźć natychmiastowego rozwiązania problemu, staję się zniecierpliwiony i poirytowany. | 1 | 2 | 3 | 4 | 5 | 6 |
| Wolę raczej podejmować decyzje szybko, niż odwlekać moment podjęcia decyzji. | 1 | 2 | 3 | 4 | 5 | 6 |
| Myślę, że zabawnie jest zmieniać plany w ostatnim momencie. | 1 | 2 | 3 | 4 | 5 | 6 |
| Bawi mnie niepewność związana z nowymi sytuacjami. | 1 | 2 | 3 | 4 | 5 | 6 |
| Moje własne otoczenie jest nieuporządkowane i niezorganizowane. | 1 | 2 | 3 | 4 | 5 | 6 |

1 - zdecydowanie nie zgadzam się

2 - nie zgadzam się

3 - raczej nie zgadzam się

4 - raczej się zgadzam

5 - zgadzam się

6 - zdecydowanie się zgadzam

| Nawet jeśli mam dużo czasu na podjęcie decyzji, odczuwam presję, aby podjąć ją szybko. | 1 | 2 | 3 | 4 | 5 | 6 |
| --- | --- | --- | --- | --- | --- | --- |
| Wierzę, że najważniejsze cechy studenta to zdyscyplinowanie i zorganizowanie. | 1 | 2 | 3 | 4 | 5 | 6 |
| Przyglądając się większości sytuacji konfliktowych potrafię zwykle dostrzec racje obu stron. | 1 | 2 | 3 | 4 | 5 | 6 |
| Unikam przebywania wśród ludzi, którzy są zdolni do nieoczekiwanych działań. | 1 | 2 | 3 | 4 | 5 | 6 |
| Preferuję kontakty ze znanymi sobie ludźmi, gdyż wiem, czego mogę się po nich spodziewać. | 1 | 2 | 3 | 4 | 5 | 6 |
| Dążę do tego, by zawsze wiedzieć, co ludzie myślą na dany temat. | 1 | 2 | 3 | 4 | 5 | 6 |
| Myślę, że lepiej uczę się w sytuacji, w której brak jasno określonych celów i wymagań. | 1 | 2 | 3 | 4 | 5 | 6 |
| Czuję dyskomfort, kiedy czyjeś stwierdzenie nasuwa wiele różnych interpretacji. | 1 | 2 | 3 | 4 | 5 | 6 |
| Dopiero ustalenie spójnych reguł umożliwia mi cieszenie się życiem. | 1 | 2 | 3 | 4 | 5 | 6 |
| Cenię sobie zorganizowany styl życia. | 1 | 2 | 3 | 4 | 5 | 6 |
| Lubię mieć miejsce dla wszystkiego i wszystko na swoim miejscu. | 1 | 2 | 3 | 4 | 5 | 6 |
| Czuję dyskomfort, gdy czyjeś czyny lub intencje są dla mnie niejasne. | 1 | 2 | 3 | 4 | 5 | 6 |
| Często, kiedy staram się rozwiązać jakiś problem, dostrzegam tak wiele różnych możliwości rozwiązań, że tracę pewność siebie. | 1 | 2 | 3 | 4 | 5 | 6 |
| Zwykle dostrzegam wiele możliwych rozwiązań problemu, przed którym stoję. | 1 | 2 | 3 | 4 | 5 | 6 |
| Unikam sytuacji, których konsekwencji nie da się przewidzieć. | 1 | 2 | 3 | 4 | 5 | 6 |
| Odczuwam potrzebę pospiesznego podejmowania decyzji, nawet jeśli nie ma ku temu powodu. | 1 | 2 | 3 | 4 | 5 | 6 |
