# Supplementary material for: Need for cognitive closure predicts preference for similar others and reduced diversity in social networks
Source: Sci Rep. 2026 Jan 16;16:5582. doi: 10.1038/s41598-026-36288-6 (PMC12891588; doi:10.1038/s41598-026-36288-6)
Supplement: Supplementary file 6 — Supplementary Material 6 [file 41598_2026_36288_MOESM6_ESM.docx]

**Supplementary Material 6**

**Instrument licensing and permissions**

All instruments were administered for non-commercial academic research.

Except for the STAI (administered under an institutional research license from Mind Garden, Inc.), all questionnaires are either open for academic use or were developed by the authors. Full details are provided below.

**Table 1.** Instrument licensing and permissions.

| **Instrument** | **Original source / adaptation** | **Copyright holder** | **Research-use status** | **Permission required** | **Action taken in this study** |
| --- | --- | --- | --- | --- | --- |
| **Need for Cognitive Closure Scale (NFC)** | Webster & Kruglanski (1994); Polish adaptation: Kossowska (2003) | Authors (1994); open academic use | Freely available for non-commercial research; adaptation licensed for research use | No | Used under open academic license; cited both sources |
| **Short Need for Cognitive Closure Scale (NFC)** | Roets & Van Hiel (2007); Polish adaptation: Kossowska et al. (2012) | Authors (2007); open academic use | Freely available for non-commercial research; adaptation licensed for research use | No | Used under open academic license; cited both sources |
| **Heterophilous Interaction Questionnaire** | Developed by Growiec (2015) | Author (2015) | Open academic instrument; published in previous research by first author | No | Used with citation to Growiec (2015); open academic use confirmed |
| **Self-Esteem Scale (SES)** | Rosenberg (1965) | University of Maryland / Public domain for research use | Freely available for non-commercial academic research and teaching | No | Used with citation to Rosenberg (1965); open academic use confirmed |
| **Positive and Negative Affect Schedule (PANAS)** | Watson, Clark & Tellegen (1988) | American Psychological Association (APA) | APA permits free non-commercial research use with citation | No | Used under APA’s non-commercial research policy |
| **State-Trait Anxiety Inventory (STAI)** | Spielberger et al. (1983) | Mind Garden Inc. / Charles D. Spielberger | Commercial instrument; research use under institutional license | No (only needed when reproducing items) | Administered under university research license from Mind Garden Inc.; no items reproduced |
| **Experimental scenarios  (Study 5)** | Developed by authors for this project | Authors (2025) | Original, open materials on OSF | No | Shared openly on OSF repository |
