# Supplementary material for: Need for cognitive closure predicts preference for similar others and reduced diversity in social networks
Source: Sci Rep. 2026 Jan 16;16:5582. doi: 10.1038/s41598-026-36288-6 (PMC12891588; doi:10.1038/s41598-026-36288-6)
Supplement: Supplementary file 7 — Supplementary Material 7 [file 41598_2026_36288_MOESM7_ESM.docx]

**Supplementary Material 7**

**Table 1.** Study 5: Sample demographic characteristics.

| **Variable** | **Response options (coding)** | **Distribution (n)** | **Interpretation** |
| --- | --- | --- | --- |
| **Age** | 1 = 18–24; 2 = 25–32; 3 = 33–40; 4 = 41–50; 5 = 51–60; 6 = 61+ | 28 / 38 / 45 / 50 / 43 / 82 | Broad age distribution. |
| **Gender** | 1 = Female; 2 = Male; 3 = Other | 146 / 139 / 1 | Balanced gender representation. |
| **Nationality** | 1 = Polish; 2 = Other | 284 / 2 | 99% Polish; minimal national diversity. |
| **Sexual orientation** | 1 = Heterosexual; 2 = Homosexual; 3 = Bisexual; 4 = Other | 260 / 10 / 10 / 5 | Predominantly heterosexual (≈90%). |
| **Skin color** | 1 = Light/white; 2 = Medium (olive/tan); 3 = Dark (light brown); 4 = Very dark (dark brown/black) | 246 / 37 / 3 / 0 | Highly homogeneous, almost all light-skinned. |
| **Religion (affiliation)** | 1 = Catholic; 2 = Orthodox; 3 = Protestant; 4 = Muslim; 5 = Jewish; 6 = Buddhist; 7 = Other; 8 = Non-religious | 252 / 1 / 0 / 1 / 0 / 0 / 5 / 27 | Vast majority Catholic (≈90%). |
| **Religious attitude** | 1 = Atheist; 2 = Agnostic; 3 = Religion is not important; 4 = Religion somewhat important; 5 = Religion very important | 28 / 11 / 96 / 96 / 55 | Moderate to high religiosity in most participants. |
| **Material situation** | 1 = Poor (basic needs unmet); 2 = Fair (must control every expense); 3 = Average (no financial problems); 4 = Good (comfortable with savings); 5 = Very good (luxurious) | 19 / 66 / 131 / 67 / 3 | Most report average or good financial status. |
| **Political orientation** | 1 = PiS; 2 = PSL; 3 = SLD; 4 = Confederation; 5 = KO; 6 = Did not vote | 85 / 7 / 19 / 20 / 80 / 75 | Diverse political representation across spectrum. |
