# Supplementary material for: Need for cognitive closure predicts preference for similar others and reduced diversity in social networks
Source: Sci Rep. 2026 Jan 16;16:5582. doi: 10.1038/s41598-026-36288-6 (PMC12891588; doi:10.1038/s41598-026-36288-6)
Supplement: Supplementary file 8 — Supplementary Material 8 [file 41598_2026_36288_MOESM8_ESM.docx]

**Supplementary Material 8**

**Table 1.** *Simple slopes for the effect of experimental condition (uncertainty vs. control) on willingness to interact across levels of similarity and Need for Cognitive Closure (NFC).*

| **NFC Level** | **Similarity Level** | **Est.** | **SE** | ***t*** | ***p*** |
| --- | --- | --- | --- | --- | --- |
| –1 SD (Low NFC) | –1 SD | 0.15 | 0.14 | 1.11 | .27 |
| –1 SD (Low NFC) | Mean | 0.17 | 0.13 | 1.31 | .19 |
| –1 SD (Low NFC) | +1 SD | 0.18 | 0.14 | 1.28 | .20 |
| Mean NFC | –1 SD | 0.07 | 0.10 | 0.75 | .45 |
| Mean NFC | Mean | 0.17 | 0.09 | 1.90 | .06 |
| **Mean NFC** | **+1 SD** | **0.26** | **0.10** | **2.72** | **.01*** |
| +1 SD (High NFC) | –1 SD | –0.01 | 0.14 | –0.06 | .95 |
| +1 SD (High NFC) | Mean | 0.17 | 0.13 | 1.37 | .17 |
| **+1 SD (High NFC)** | **+1 SD** | **0.35** | **0.14** | **2.54** | **.01*** |

*Note.* Bold indicates *p* < .05.
