# Supplementary material for: Need for cognitive closure predicts preference for similar others and reduced diversity in social networks
Source: Sci Rep. 2026 Jan 16;16:5582. doi: 10.1038/s41598-026-36288-6 (PMC12891588; doi:10.1038/s41598-026-36288-6)
Supplement: Supplementary file 9 — Supplementary Material 9 [file 41598_2026_36288_MOESM9_ESM.docx]

**Supplementary Material 9**

**Supplementary Analyses: Controlling for Political Ideology**

To assess whether our findings were influenced by participants’ political ideology, we included political orientation as a covariate in all multilevel models. Participants reported which party they voted for in the 2019 Polish parliamentary elections. Responses were recoded into a three-level categorical variable reflecting broad ideological alignment: Right (coded as reference category): PiS (*Prawo
i Sprawiedliwość*) and *Konfederacja (KWiN*), Center: PSL (*Polskie Stronnictwo Ludowe*) and KO (*Koalicja Obywatelska*), Left: SLD (*Sojusz Lewicy Demokratycznej*).

Participants who reported not voting were excluded from these models (final *n* = 171). All models included fixed effects for similarity (demographic or psychological), experimental condition (uncertainty vs. control), need for cognitive closure (NFC), and political orientation, with a random intercept for participant. Full model results are presented in **Table 1**.

**Results and Interpretation**

For models examining the effect of similarity and condition (without NFC):

- **Demographic similarity:** The main effect of similarity and the similarity × condition interaction remained significant or marginally significant (*p* = .055), suggesting that under uncertainty, participants prefer demographically similar others. The slight increase in *p*-value likely reflects reduced power due to excluding non-voters, as the effect estimates remained consistent.
- **Psychological similarity:** The main effect of similarity on willingness to interact was preserved. However, the main effect of condition was no longer significant, indicating a weaker or less robust moderation by uncertainty in this subset.

For models including also NFC:

- **Demographic similarity:** The critical three-way interaction (similarity × condition × NFC) remained significant, confirming that individuals high in NFC showed stronger preferences for similar others under uncertainty—even when controlling for political ideology.
- **Psychological similarity:** Interestingly, the previously non-significant three-way interaction became statistically significant in this model. This suggests that political orientation may have suppressed or obscured the moderating effect of NFC on responses to psychological similarity under uncertainty.

**Conclusion**

The inclusion of political ideology as a covariate did not meaningfully alter our main findings. The key interactions between similarity, uncertainty, and NFC remained significant (or nearly so). This suggests that the effects observed in our studies are not driven by participants’ ideological alignment. However, the emergence of a significant three-way interaction for psychological similarity indicates that future work could explore how ideological orientation interacts with motivational needs (like NFC) to shape social preferences. This effect may merit further investigation in studies specifically powered to test such moderation.

| **Table 1.** Results of linear mixed-effects models controlling for political orientation. | | | | | | | | | | | | |
| --- | --- | --- | --- | --- | --- | --- | --- | --- | --- | --- | --- | --- |
|  | **Demographic Similarity** | | | **Psychological Similarity** | | | **Demographic Similarity × NFC** | | | **Psychological Similarity × NFC** | | |
| *Predictors* | *Est.* | *SE* | *p* | *Est.* | *SE* | *p* | *Est.* | *SE* | *p* | *Est.* | *SE* | *p* |
| (Intercept) | 0.05 | 0.08 | 0.572 | 0.06 | 0.08 | 0.409 | 0.06 | 0.08 | 0.496 | 0.07 | 0.08 | 0.375 |
| Demographic similarity | 0.14 | 0.03 | **<0.001** |  |  |  | 0.13 | 0.03 | **<0.001** |  |  |  |
| Condition: Uncertainty | 0.16 | 0.10 | 0.102 | 0.13 | 0.09 | 0.142 | 0.15 | 0.10 | 0.125 | 0.13 | 0.09 | 0.165 |
| Political ideology: Center vs. right | -0.19 | 0.10 | 0.068 | -0.11 | 0.10 | 0.248 | -0.19 | 0.10 | 0.073 | -0.10 | 0.10 | 0.274 |
| Political ideology: Left vs. right | -0.11 | 0.17 | 0.537 | -0.07 | 0.16 | 0.680 | -0.12 | 0.17 | 0.471 | -0.09 | 0.16 | 0.589 |
| Demographic similarity × Condition | 0.09 | 0.05 | **0.055** |  |  |  | 0.09 | 0.05 | **0.048** |  |  |  |
| Psychological similarity |  |  |  | 0.26 | 0.03 | **<0.001** |  |  |  | 0.25 | 0.03 | **<0.001** |
| Psychological similarity × Condition |  |  |  | 0.02 | 0.04 | 0.580 |  |  |  | 0.03 | 0.04 | 0.486 |
| NFC |  |  |  |  |  |  | 0.05 | 0.07 | 0.482 | 0.04 | 0.07 | 0.541 |
| Demographic similarity × NFC |  |  |  |  |  |  | -0.08 | 0.03 | **0.012** |  |  |  |
| NFC × Condition |  |  |  |  |  |  | -0.14 | 0.10 | 0.170 | -0.19 | 0.10 | **0.048** |
| Demographic similarity × Condition × NFC |  |  |  |  |  |  | 0.10 | 0.05 | **0.050** |  |  |  |
| Psychological similarity × NFC |  |  |  |  |  |  |  |  |  | -0.02 | 0.03 | 0.439 |
| Psychological similarity × Condition × NFC |  |  |  |  |  |  |  |  |  | 0.09 | 0.04 | **0.048** |
| **Random Effects** | | | | | | | | | | | | |
| σ^2^ | 0.52 | | | 0.47 | | | 0.51 | | | 0.46 | | |
| τ_00_ | 0.34 | | | 0.29 | | | 0.35 | | | 0.29 | | |
| ICC | 0.40 | | | 0.38 | | | 0.40 | | | 0.38 | | |
| Marginal R^2^ / Conditional R^2^ | 0.053 / 0.430 | | | 0.100 / 0.444 | | | 0.063 / 0.439 | | | 0.113 / 0.451 | | |
