# Supplementary material for: Need for cognitive closure predicts preference for similar others and reduced diversity in social networks
Source: Sci Rep. 2026 Jan 16;16:5582. doi: 10.1038/s41598-026-36288-6 (PMC12891588; doi:10.1038/s41598-026-36288-6)
Supplement: Supplementary file 10 — Supplementary Material 10 [file 41598_2026_36288_MOESM10_ESM.docx]

**Supplementary Material 10**

**Range of responses for willingness to interact along four items (“meet,” “talk,” “write,” “spend time”)**

Each item was rated on a 7-point scale (1 = not at all, 7 = very much)

Table 1.

| **Dimension** | **Mean** | **SD** | **Min** | **Max** |
| --- | --- | --- | --- | --- |
| Meet | 5,04 | 1,88 | 1 | 7 |
| Talk | 4,94 | 1,92 | 1 | 7 |
| Write | 5,04 | 1,87 | 1 | 7 |
| Spend time | 4,45 | 2,1 | 1 | 7 |
